# The Gait Deviation Index Is Associated with Hip Muscle Strength and Patient-Reported Outcome in Patients with Severe Hip Osteoarthritis—A Cross-Sectional Study

**DOI:** 10.1371/journal.pone.0153177

**Published:** 2016-04-11

**Authors:** Signe Rosenlund, Anders Holsgaard-Larsen, Søren Overgaard, Carsten Jensen

**Affiliations:** 1 Department of Orthopaedic Surgery and Traumatology, Odense University Hospital Institute of Clinical Research, University of Southern Denmark, Odense C, Denmark; 2 Department of Orthopaedic Surgery and Traumatology, Køge Hospital, Køge, Denmark; Rutgers University -New Jersey Medical School, UNITED STATES

## Abstract

**Background:**

The Gait Deviation Index summarizes overall gait ‘quality’, based on kinematic data from a 3-dimensional gait analysis. However, it is unknown which clinical outcomes may affect the Gait Deviation Index in patients with primary hip osteoarthritis. The aim of this study was to investigate associations between Gait Deviation Index as a measure of gait ‘quality’ and hip muscle strength and between Gait Deviation Index and patient-reported outcomes in patients with primary hip osteoarthritis.

**Method:**

Forty-seven patients (34 males), aged 61.1 ± 6.7 years, with BMI 27.3 ± 3.4 (kg/m^2^) and with severe primary hip osteoarthritis underwent 3-dimensional gait analysis. Mean Gait Deviation Index, pain after walking and maximal isometric hip muscle strength (flexor, extensor, and abductor) were recorded. All patients completed the ‘Physical Function Short-form of the Hip disability and Osteoarthritis Outcome Score (HOOS-Physical Function) and the Hip disability and Osteoarthritis Outcome Score subscales for pain (HOOS-Pain) and quality-of-life (HOOS-QOL).

**Results:**

Mean Gait Deviation Index was positively associated with hip abduction strength (p<0.01, *r* = 0.40), hip flexion strength (p = 0.01, *r* = 0.37), HOOS-Physical Function (p<0.01, *r* = 0.41) HOOS-QOL (p<0.01, *r* = 0.41), and negatively associated with HOOS-Pain after walking (p<0.01, *r* = -0.45). Adjusting the analysis for walking speed did not affect the association.

**Conclusion:**

Patients with the strongest hip abductor and hip flexor muscles had the best gait ‘quality’. Furthermore, patients with higher physical function, quality of life scores and lower pain levels demonstrated better gait ‘quality’. These findings indicate that interventions aimed at improving hip muscle strength and pain management may to a moderate degree improve the overall gait ‘quality’ in patients with primary hip OA.

## Introduction

Hip osteoarthritis (OA) is a leading cause of disability worldwide [1]. Patients with hip OA walk more slowly, walk with altered gait pattern characteristics [2, 3] that are often reported as limping gait [4] and have reduced hip muscle strength compared with healthy controls [5–8]. These impairments may also be reflected in patient-reported outcomes (PROs) [9, 10]. A study by Amlie et al. 2014 showed that hip OA patients with self-reported limping gait after total hip arthroplasty (THA) scored 17 to 35 points worse in PROs on all the subscales of Hip disability and Osteoarthritis Outcome Score (HOOS) (0 = extreme symptoms to 100 = no symptoms) compared with patients who reported no limping [11]. This indicates that the walking pattern is important to patients post-operatively and may also be important prior to surgery. However, limping gait is poorly defined and is often based on a subjective analysis performed by the patient or the clinician.

Three-dimensional gait analysis (3DGA) is an objective and detailed analysis of the walking pattern, with the possibility to measure a large number of discrete variables. Discrete variables are often measured at one time-point in the gait cycle, for example hip or knee extension at terminal stance or peak hip flexion and extension. However these discrete variables lack the ability to evaluate the overall gait pattern or gait ‘quality’ throughout the entire gait cycle [12]. Therefore, there is an increasing interest in implementing a summary index that describes the overall gait ‘quality’ expressed as the degree of deviation from normalcy. The most common overall gait index used to date is the Gillette Index, but several limitations have been raised. The selection of variables included in the Gillette Index was largely based on gait deviation experienced in children with cerebral palsy and only the characteristic discrete time-points in the gait cycle were included [13]. In contrast, the Gait Deviation Index (GDI) was developed with the intention of providing an overall gait ‘quality’ index [14]. GDI summarizes the lower limb kinematic of nine variables for each limb throughout the entire gait cycle into one single value that describes 98% of the variation in gait. The nine variables included in the GDI are kinematics from: pelvic and hip in sagittal, frontal and transversal plane, knee and ankle in the sagittal plane and foot progression [14]. The GDI expresses the degree of gait pathology in patients compared with a healthy reference group [14]. This removes the subjectivity in the choice of discrete parameters. Furthermore, the selection of the parameters for the Gillette Index was specific for children with cerebral palsy, whereas the GDI is considered a general measure of the overall gait pathology or gait ‘quality’ in humans [14]. The GDI has been applied on different patient groups ranging from patients with cerebral palsy [15–18] to patients with hip OA [19, 20], patients with rheumatoid arthritis [21] and patients with Parkinson’s Disease [22]. Research into associations between the GDI as a measure of gait ‘quality’ and validated clinically important outcomes will improve understanding of the clinical utility and application of the GDI in research and in the clinical context. This has, to our knowledge, only been performed in cerebral palsy and rheumatoid arthritis patients [18, 21, 23].

The purpose of this study was to investigate potential associations between gait ‘quality’ measured by the GDI, hip muscle strength and PROs in patients with severe primary hip OA. We hypothesized that low hip muscle strength, high pain level and impaired self-reported physical function and quality of life would be associated with reduced GDI scores. The results from this study will provide a better understanding of the factors influencing the overall gait ‘quality’ and the way in which targeted rehabilitation may improve gait ‘quality’ for hip OA patients.

## Materials and Methods

### Design

This study was a cross-sectional analysis of explorative pre-operative data from a subsample of patients included in a randomized controlled trial. The trial is registered at ClinicalTrials.gov. No.:NCT01616667 and described in detail elsewhere [24].

### Participants

Forty-seven patients were recruited from the outpatient clinic at the Department of Orthopaedic Surgery and Traumatology, Odense University Hospital and Svendborg Hospital (SvB), Denmark between May 2012 and May 2014. The inclusion criteria were patients aged 45–70 years with primary unilateral hip OA, scheduled for cementless THA. Patients were excluded if they had had prior THA or major lower limb surgery, BMI>35 (kg/m^2^) (due to difficulties with gait analysis marker placement), if they were unable to walk without walking aid of any kind, if they had any neurological or medical disease compromising their walking ability, inability to read and understand Danish, or if they declined to participate. All patients were clinically and radio-graphically evaluated and diagnosed with primary OA and scheduled for THA. A convenience sample of 20 able-bodied adults aged 45 to 70 years was recruited to provide a reference group on kinematic data.

The patients’ characteristics are summarized in Table 1. Forty-seven patients and 20 able-bodied participants completed the evaluation with 3DGA and hip muscle iMVC. The patient group included 34 males and 13 females. They had a mean age of 61.1 ± 6.7 years and a mean BMI of 27.3 ± 3.4 (kg/m2).

**Table 1:** Characteristics of hip OA patients and able-bodied.

Characteristics	Hip OA-patients (n = 47)	Able-bodied (n = 20)	P-value
**Age (years), mean ± SD**	61.1 ± 6.7	56.9 ± 7.1	0.02^a^
**Male (%)**	34 (72)	9 (45)	0.03^b^
**BMI (kg/m**^**2**^**), mean ± SD**	27.3 ± 3.4	25.6 ± 2.9	0.05^a^
**Self-selected walking speed (m/s), mean ± SD**	1.12 ± 0.18	1.33 ± 0.14	< 0.01^a^
**Affected side (Right)**	25	Not relevant	-

Abbreviations: OA = Osteoarthritis; SD = standard deviation; BMI = body mass index

^a^ Independent unpaired t-test with equal variance

^b^ Pearson’s chi2 test

The able-bodied participants (55% female) had a mean age of 59.6 ±7.1 years and mean BMI of 25.6 ±2.9 (kg/m2), Table 1. The able-bodied participants walked, as expected, significantly faster than the OA patients (p < 0.01).

The trial complied with the Declaration of Helsinki. It was approved by the Danish Data Protection Agency and The Danish Regional Committee on Biomedical Research Ethics (Southern Denmark), Project-ID S-20120009. A written and orally informed consent was collected prior to the inclusion of all participants.

### Gait data collection

3DGA data were collected using a six-camera motion capture system (Vicon MX03, Oxford, UK) and further processed in Nexus 1.7–8 [25, 26]. Prior to the gait analysis the leg length, pelvic, knee and angle width were measured. These measurements were used to estimate joint rotation centres for each individual. A total of 16 markers were placed on anatomical landmarks on the pelvic, thigh, knee and foot according the standardized Plug-in-Gait marker set model [27]. All the participants walked barefooted at self-selected walking speed on a 10 meter long walking corridor. The patients and able-bodied participants completed an identical motion capture protocol. We captured five separate gait trials for each participant as recommended by Laroche et al. 2011 [28]. To avoid acceleration and deceleration only the stride in the centre of the capturing volume for each trial (five left and five right) were analysed.

### Maximal hip muscle strength

The muscle strength of the hip abductor, hip flexor and hip extensor muscles was measured with three consecutive isometric maximal voluntary contractions (iMVC) for each muscle group. Maximal hip muscle strength was recorded in the upright standing position, according to the protocol described by Jensen et al. [29]. For each muscle group, one submaximal test contraction (for familiarization) and three maximal contractions were performed. The participants received both visual feedback on a pc-monitor (in the form of a graphical plot of the force over time) and verbal feedback during each test contraction. To avoid a systematic learning bias, two dice randomizations were used to determine first the starting leg and second the sequence of muscle groups to be tested. The two data collectors performed a visual inspection (face validity) together of the contraction curves to ensure no pretension using a custom-made MATLAB^®^ program (MathWorks, Natick, MA, US). The contraction with the highest peak moment (Nm) was selected and normalized according to body mass (Nm/kg). Only iMVC performed with the affected limb was used in the current study. iMVC in the standing position has shown acceptable validity [30] and high test-retest reliability has been reported in healthy adults and patients with THA [29, 30].

### Pain assessment

Pain was measured immediately after completion of the 3DGA using an 11-point (0–10) Numeric Rating Scale (NRS); where 0 represents ‘no pain’ and 10 represents ‘the worst possible pain’. The patients were asked to assess their hip pain intensity for the affected limb immediately after walking. A second pain intensity assessment was performed immediately after the last iMVC contraction. The NRS scores for the affected limbs recorded after walking were used in the association analyses. The NRS has been shown to be both valid and reliable in geriatric patients [31].

### Patient-reported outcome measures

We used the Hip disability and Osteoarthritis Outcome Score–Physical Function Short-form together with its subscales of Pain and Quality Of Life in HOOS 2.0 (HOOS-Pain and HOOS-QOL) to measure physical function, pain and quality of life, respectively. The total score for each of the three subscales ranges from 0 points (extreme symptoms) to 100 points (no symptoms). HOOS-Physical Function has shown good validity when compared with the HOOS subscale for Activity of Daily Living and high responsiveness in THA patients [32]. HOOS 2.0 has shown high validity, reliability and responsiveness in patients diagnosed with hip OA [33]. The patients completed the Danish version of the questionnaire that has been validated for trans-cultural adaptation [34].

### Data processing

The GDI is calculated for each limb using nine kinematic variables collected during 3DGA- namely, all three planes from the pelvis and hip, the sagittal plane from the knee and ankle and the foot progression [14]. First, the GDI scores for the 20 able-bodied participants were calculated according to the instructions described by its authors using the electronic addendum (an Microsoft Excel file) provided with the original paper by Schwartz and Rozumalski 2008 [14]. We validated our reference group data based on the original reference data of typically developing children [14] and found that our reference group had a mean GDI (mGDI) of 94.4 ± 7.3 Next, we replaced the original reference data in the electronic addendum with our reference group data–which *per se* had an index value of 100. Finally, we calculated the GDI scores for the hip OA patients against our reference group data. mGDI scores based on five trials were calculated. We only used the mGDI of the affected limbs in the analyses. A GDI score of 100 or above represents no gait pathology. Each 10-point decrement from 100 indicates approximately one standard deviation from the normal gait pattern [14]. GDI has shown good validity in children with cerebral palsy [35] and excellent inter-trial and intra-rater reliability [16, 21].

### Statistical Analysis

Data were checked for completeness and normality (Q-Q plot). A one-sample t-test was used to test the difference between the patients’ mGDI and the reference group’s GDI of 100 points.

**Fig 1:**
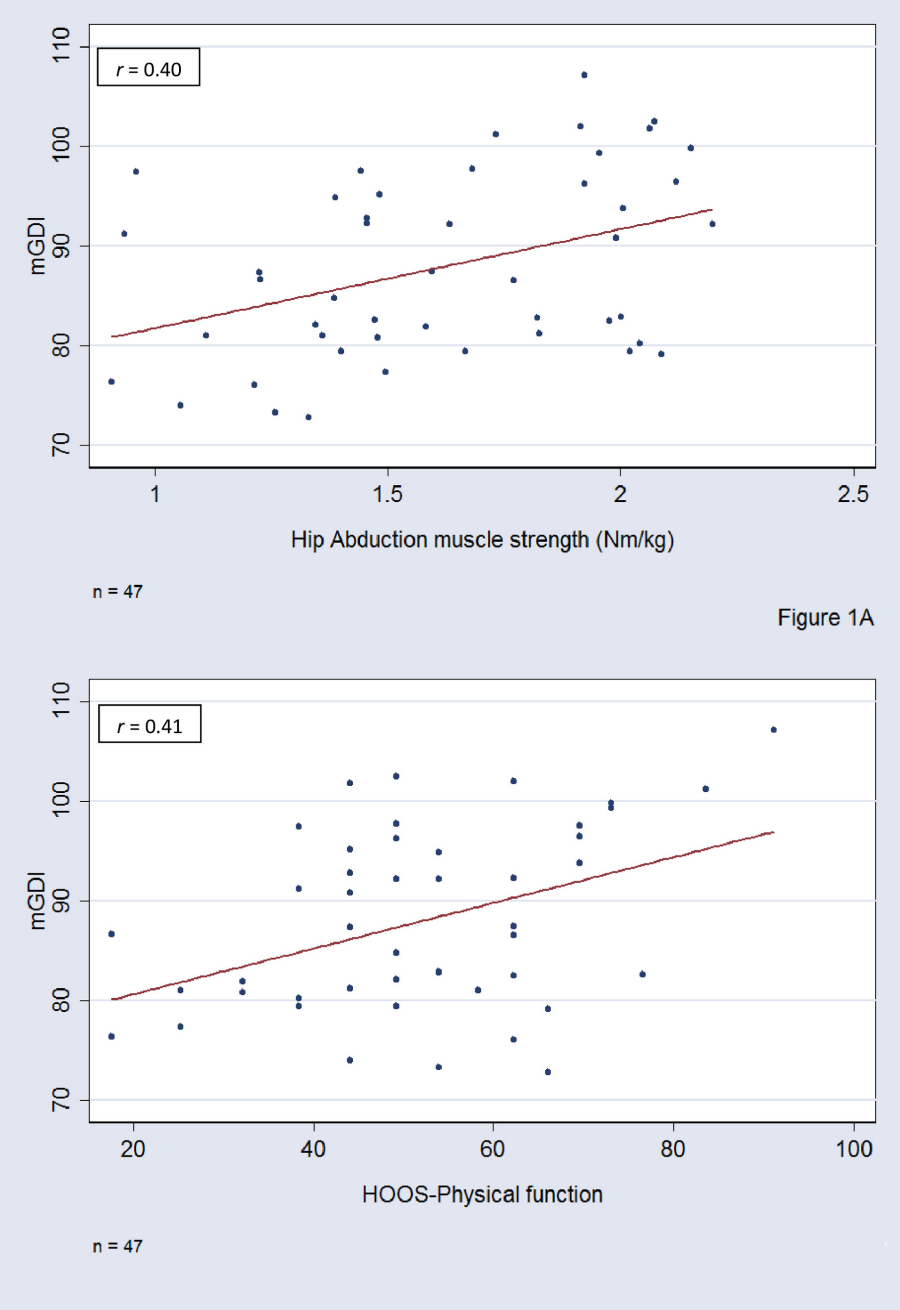
A-B. Representative examples of the association between mGDI and clinical outcomes.

We used Pearson’s correlation to investigate the associations between mGDI and continuous variables (iMVC in hip flexion, extension and abduction, HOOS-Physical Function, HOOS-Pain and HOOS-QOL). Spearman correlation was used to investigate the association between mGDI and pain measured by the NRS (ordinal data). Preliminary analyses were performed to ensure all correlation analysis assumptions were fulfilled and data were checked for linearity and outliers (Fig 1A and 1B. representative examples). Further, multiple linear regression was used to investigate the associations adjusting for walking speed. Walking speed has an influence on the kinematic in the lower limb [36, 37] and it has also been shown that the GDI may be affected by gait speed [38]. The correlation coefficients (*r-value*) and the determination coefficient (*R*^*2*^) were reported. The correlation coefficients were interpreted according to Dancey and Reidy [39]: an *r-value* of 0.1–0.3 was regarded as weak; 0.4–0.6 as moderate; 0.7–0.9 as strong and 1 as perfect correlation. The statistical analyses were performed using Stata 13.1 (Stata Corp LP, Brownsville, TX, US) and we used a 0.05 significance level.

## Results

The descriptive clinical data are summarized in Table 2. Hip OA patients had moderately decreased mGDI scores on the affected limb compared with the reference group (87.9 ± 9.1 vs. 100, p<0.01).

**Table 2:** Descriptive outcome data for the patients: mean Gait Deviation Index, hip muscle strength and pain for the affected limb and patient-reported outcome measures.

Variables		Hip OA-patients (n = 47)
**Mean GDI (0 ≥ 100) in affected limb, mean ± SD**		
	GDI	87.9 ± 9.1
**Hip muscle strength in affected limb, mean ± SD**		
	Hip Extension (Nm/kg)	2.28 ± 0.55
	Hip Flexion (Nm/kg)	1.32 ± 0.38
	Hip Abduction (Nm/kg)	1.62 ± 0.36
**NRS-pain (0–10) in affected limb**, **median (Q1;Q3)**		
	After walking	5 (2;6)
	Number of patients’ with NRS > 5 (%)	17 (36%)
	After muscle strength test	6 (3;8)
	Number of patients’ with NRS > 5 (%)	24 (51%)
**HOOS subscales (0–100), mean ± SD**		
	HOOS-Physical Function	51.8 ± 16.2
	HOOS-Pain	43.4 ± 15.8
	HOOS-Quality of Life	29.0 ± 14.5

Q1; Q3 represent 25^th^ and 75^th^ percentiles. Abbreviations: OA = Osteoarthritis; GDI = Gait Deviation Index; NRS = Numeric Rating Scale for pain (0 represents ‘no pain’ and 10 represents ‘the worst possible pain’); HOOS = Hip disability and Osteoarthritis Outcome Score ranges from 0 = extreme symptoms to 100 = no symptoms

Results from the unadjusted and adjusted associations between the mGDI scores and the independent clinical outcomes are summarized in Table 3. A significant, moderate association between hip abduction muscle strength and mGDI was identified (*r* = 0.40; p<0.01). We also found a significant, albeit weak, association between hip flexion muscle strength and mGDI (*r* = 0.37; p = 0.01), but no association between hip extension muscle strength and mGDI (*r* = 0.10; p = 0.50). Adjusting for walking speed did not change the significance of the association between mGDI and hip muscle strength, Table 3.

**Table 3:** Simple and adjusted associations between mGDI as the dependent variable and hip muscle strength, pain and HOOS scores as independent variables.

Independent outcome variables	Simple association*			Adjusted association**		
	*r*-value	*R*^2^	*P*-value	*r*-value	*R*^2^	*P*-value
**Hip Extension**	0.10	0.01	0.50	0.33	0.11	0.08
**Hip Flexion**	0.37	0.13	0.01	0.41	0.17	0.02
**Hip Abduction**	0.40	0.16	<0.01	0.43	0.19	0.01
**NRS-pain after walking**	-0.45	0.20	<0.01	-0.48	0.23	<0.01
**HOOS-Physical Function**	0.41	0.17	<0.01	0.45	0.20	<0.01
**HOOS-Pain**	0.18	0.03	0.23	0.35	0.12	0.06
**HOOS-Quality of Life**	0.41	0.17	<0.01	0.50	0.25	<0.01

* Continuous data analysed with Pearson’s correlation and ordinal data analysed with Spearman correlation

** Multiple linear regressions adjusting for walking speed

Abbreviations: OA = Osteoarthritis; GDI = Gait Deviation Index; NRS = Numeric Rating Scale for pain; HOOS = Hip disability and Osteoarthritis Outcome Score

We found a moderately significant negative association between pain measured by NRS immediately after walking and mGDI (*r* = -0.45; p<0.01), Table 3. Furthermore, moderately significant associations between mGDI and patient-reported physical function and quality of life (*r* = 0.41; p<0.01 and *r* = 0.41; p<0.01, respectively) were found. These moderately significant associations were also present after adjusting for walking speed, Table 3. However; no association between mGDI and HOOS-Pain was found (*r* = 0.18; p = 0.23).

## Discussion

This study investigated the potential influence of hip muscle strength, patient-reported physical function, pain and quality of life on the gait ‘quality’ of patients with hip OA.

### Hip muscle strength and gait ‘quality’

The results confirmed our hypotheses that hip OA patients who experience low hip muscle strength also walk with reduced gait ‘quality’, but to a lesser degree than anticipated. The current weak to moderate associations emphasize that reduced iMVC does not reflect the entire functional limitation of gait suggesting that factors not identified in the current study are also of importance for gait ‘quality’. However, our results are consistent with previous findings of weak to moderate associations between hip abductor muscle strength and discrete kinematic variables of gait in hip OA patients and healthy older adults [12, 40]. The GDI was measured during horizontal walking at a self-selected walking speed. This task may not be challenging enough to ensure high demand on the hip muscles in hip OA patients, which is reflected in the weak to moderate association. This may also be the reason why we did not find any association between mGDI and hip extensor muscle strength. Little strength requirements in this muscle group is needed during horizontal walking [41]. The hip muscle strength was measured isometrically in a standing position with the limb in an anatomically neutral position. However, this position only represents the position of the joint at one time point in the gait cycle. Analysis of measures of isokinetic hip muscle strength in a dynamic setup mimicking the range of motion and force-velocity during gait may supply the analysis and represent a more functional model. Our finding of a positive association between hip abductor muscle strength and mGDI is however in line with several previous studies that found hip abductor muscle strength to be important for gait function and emphasized the need for increased muscle strengthening regimens [5, 42–45]. The hip abductors are important to stabilize the pelvis in the frontal plane during the stance phase and hip abductor weakness may cause the pelvis to drop towards the swing limb [46] and thus influence the kinematic of the pelvis included in the GDI. Our results suggest that an effective rehabilitation intervention with special focus on the hip abductor muscles will, to some extent, improve the gait ‘quality’ for patients with hip OA although it only accounted for 16% of the variation in gait. Thus, this study contributed to identify adjustable physical parameters that influence the gait ‘quality’. It is essential to identify these parameters and know the strength of associations to gait when developing rehabilitation programs that intend to improve the overall gait in patients with hip OA.

### Pain and gait ‘quality’

We found a moderately negative association between gait ‘quality’ and pain measured directly after walking that explained 20% of the variation in GDI but we found no association between gait ‘quality’ and pain measured over the past week (HOOS-pain). The discrepancy in our results could be explained by the way the two pain score are collected. The NRS score is directly connected to the pain level experienced by the patient at the time of walking, whereas the items in the HOOS-pain subscale cover several situations over the past week, not only related to the activity of walking. In accordance with our result, both Behery and Foucher 2014 and Zeni et al. 2014 [10, 12] investigated the association between pain measured as the average over the last week and discrete 3DGA parameters and they found no association in patients with hip OA. Thus, our finding suggests that high pain level experienced directly in relation to gait is negatively associated with the objective measured gait ‘quality’ and that it matters at which time-point pain is measured. Our results imply that interventions aimed at improving pain management may improve gait ‘quality’ to a moderate degree in patients with severe hip OA.

### Patient-reported outcomes and gait ‘quality’

The moderate association between mGDI and self-reported physical function and quality of life is also in agreement with previous findings [10]. Associations between the Harris Hip Score (HHS) (which is a commonly used surgeon-reported outcome measure) and several specific gait variables (hip range of motion and joint moments in all three planes of the hip) before and after THA surgery have been identified [10]. An association between the HHS subscale of physical function and kinematic gait parameters, in particular, has been observed [10]. The variance not accounted for between mGDI and PROs in this study may be because self-reported physical function and actual physical performance are two different constructs, but hypothesized to have some similarities. Therefore, strong associations cannot be expected. However, our results underline that both types of measurement are important to obtain a more complete evaluation of hip OA patients [47, 48]. Further this study has shown an association between patient-reported outcome and gait ‘quality’. These findings indicate that gait ‘quality’ may be important for the well-being of patients with hip OA as it has been shown for patients with THA [11].

### Interactions of outcomes between domains

As recommended by ‘Outcome Measures in Rheumatology Clinical Trials’ [49], evaluation of both objective and patient-reported measures was performed in the current study. However, in this study, we did not investigate the associations between hip muscle strength and PROs directly, but it has previously been shown that improving the hip abductors significantly improves both pain and patient-reported physical function in people with knee OA [50] and hip OA [47, 51]. Thus, improvement of hip abductor strength would possibly improve both physical function and gait ‘quality’ in hip OA patients.

We have also investigated the interaction between different domains in accordance with WHO International Classification of Functioning, Disability and Health (ICF) classification [52]. The ICF classification specifies three domains (‘body function’, ‘activity’ and ‘participation’) that influence a person’s functioning, disability and health status and the model implies that the domains are interacting. This study covered two of the domains, namely, ‘body function’ with the measure of gait ‘quality’ and hip muscle strength and the ‘participation’ domain reflected in the PROs. We observed weak to moderate associations both within the domain of ‘body function’ (gait ‘quality’ and hip muscle strength) and between the domains (gait ‘quality’ and PROs) supporting the interaction between the domains.

### Strengths and limitations

We conducted an explorative study on a well-defined patient group with unilateral hip OA, which supports the internal validity of the study. The use of the GDI as a measure of gait ‘quality’ has advantages over more common ways of reporting the results from 3DGA. Firstly, by reporting a single composite score rather than several discrete gait variables extracted at specific time points in the gait cycle, the GDI can be used as an overall score of gait pathology. Secondly, the GDI has shown previously to be a potentially useful classification and evaluation tool in different patients [15, 18, 19, 21, 22, 53]. Furthermore, we based the GDI calculation upon 3DGA data collected on an adult abled-bodied reference group in our own laboratory instead of using the reference data from the literature based upon typically developing children [14]. Walking speed is known to influence the kinematic parameters [36, 37, 54]; however, all significant results remained significant after adjusting for walking speed, which shows robustness in our findings.

Finally, the evaluation included the valid and reliable HOOS-questionnaire, pain measured directly related to the gait analysis session and measurement of hip muscle strength. Thus, our results may be one step towards providing ‘validity’ to the use of the GDI in hip OA patients.

However, limitations of the current study should be mentioned. Firstly, limitations with regard to 3DGA methodology made strict inclusion criteria necessary [24]. This can affect the external validity of the study. Secondly, the GDI score does not provide any information of the underlying cause of the deviations from normal gait and does not include tempo-spatial parameters or kinetic data. Thirdly, pain during testing may limit the measurements of iMVC, because pain potentially reduces the patient’s effort in reaching maximal strength [55]. As twenty-four (51%) patients in our study had NRS pain scores of five or above after the iMVC test, (Table 2), this may have affected the association between the GDI and hip muscle strength. Finally, it must be emphasized that this is an explorative cross-sectional study where clear causal relationships cannot be established. A prospective intervention study investigating if improving the maximal hip muscle strength improves the gait ‘quality’, as this cross-sectional study implies, is therefore warranted.

## Conclusion

Better hip flexor and hip abductor muscle strength were weakly to moderately associated with a better gait ‘quality’ in patients with hip osteoarthritis. Furthermore, patients with higher patient-reported physical function, quality of life and lower pain levels demonstrated better gait ‘quality’. Interventions aimed at improving hip muscle strength, especially hip abduction strength, and appropriate pain management may to a moderate degree improve the gait ‘quality’ in patients with severe hip osteoarthritis. This, however, needs to be confirmed in controlled intervention studies.

## Supporting Information

S1 File Gait Deviation Index and Hip Muscle Strength data. (XLSX)
